# The STAR care pathway for patients with chronic pain after total knee replacement: four-year follow-up of a randomised controlled trial

**DOI:** 10.1186/s12891-023-07099-x

**Published:** 2023-12-16

**Authors:** Wendy Bertram, Vikki Wylde, Nick Howells, Beverly Shirkey, Tim J. Peters, Liang Zhu, Sian Noble, Emma Johnson, Andrew D. Beswick, Andrew Moore, Julie Bruce, David Walsh, Christopher Eccleston, Rachael Gooberman-Hill

**Affiliations:** 1 Bristol Medical School, University of Bristol, Bristol, UK; 2 National Institute for Health and Care Research Bristol Biomedical Research Centre, University Hospitals Bristol and Weston NHS Foundation Trust and the University of Bristol, Bristol, UK; 3 North Bristol NHS Trust, Bristol, UK; 4 Bristol Trials Centre, University of Bristol, Bristol, UK; 5 Bristol Dental School, University of Bristol, Bristol, UK; 6 Warwick Clinical Trials Unit, Division of Health Sciences, University of Warwick, Warwick, UK; 7 Pain Centre, National Institute for Health Research Nottingham Biomedical Research Centre, Versus Arthritis, University of Nottingham and Sherwood Forest Hospitals NHS Foundation Trust, Nottingham, UK; 8 Centre for Pain Research, The University of Bath, Bath, UK

**Keywords:** Osteoarthritis, Total knee replacement, Care pathway, Chronic post-surgical pain

## Abstract

**Background:**

The Support and Treatment After Replacement (STAR) care pathway is a clinically important and cost-effective intervention found to improve pain outcomes over one year for people with chronic pain three months after total knee replacement (TKR). We followed up STAR trial participants to evaluate the longer-term clinical- and cost-effectiveness of this care pathway.

**Methods:**

Participants who remained enrolled on the trial at one year were contacted by post at a median of four years after randomisation and invited to complete a questionnaire comprising the same outcomes collected during the trial. We captured pain (co-primary outcome using the Brief Pain Inventory (BPI) pain severity and interference scales; scored 0–10, best to worst), function, neuropathic characteristics, emotional aspects of pain, health-related quality of life, and satisfaction. Electronic hospital informatics data on hospital resource use for the period of one to four years post-randomisation were collected from participating hospital sites. The economic evaluation took an National Health Service (NHS) secondary care perspective, with a four-year time horizon.

**Results:**

Overall, 226/337 (67%) of participants returned completed follow-up questionnaires, yielding adjusted between-group differences in BPI means of -0.42 (95% confidence interval, CI (-1.07, 0.23); *p* = 0.20) for pain severity and − 0.64 (95% CI -1.41, 0.12); *p* = 0.10) for pain interference. Analysis using a multiple imputed data set (n = 337) showed an incremental net monetary benefit in favour of the STAR care pathway of £3,525 (95% CI -£990 to £8,039) at a £20,000/QALY willingness-to-pay threshold, leading to a probability that the intervention was cost-effective of 0.94.

**Conclusions:**

The magnitude of the longer-term benefits of the STAR care pathway are uncertain due to attrition of trial participants; however, there is a suggestion of some degree of sustained clinical benefit at four years. The care pathway remained cost-effective at four years.

**Trial registration:**

ISRCTN: 92,545,361.

**Supplementary Information:**

The online version contains supplementary material available at 10.1186/s12891-023-07099-x.

## Background

Treatment of osteoarthritis with total knee replacement (TKR) aims to reduce pain and functional limitations. Over 100,000 primary TKRs are performed annually by the National Health Service (NHS) in the United Kindgdom [1, 2]. Despite good outcomes for many, some patients report chronic pain after TKR, which is defined as pain that ‘develops after a surgical procedure or increases in intensity after the surgical procedure [3]. In our systematic review of studies involving patients with TKR, 10 to 34% of patients reported moderate to severe chronic post-surgical pain [4]. More recent studies report similar findings, with rates of chronic pain ranging from 15 to 29% [5–8].

We designed a care pathway intervention for people with early chronic pain after TKR and evaluated it in a multi-centre pragmatic, open randomised controlled trial [9–13]. The Support and Treatment After Replacement (STAR) care pathway involves comprehensive screening to identify those in pain three months after TKR, a one-hour assessment to identify the potential cause(s) of the pain, onward referral to existing services for appropriate treatment, and telephone follow-up over one year. At one year after randomisation, participants randomised to the STAR care pathway had better pain outcomes and fewer inpatient hospital stays compared with those who had usual care alone. The trial demonstrated that the STAR care pathway is a clinically important and cost-effective intervention that improved pain over one year for people identified as having chronic pain three months after TKR [13]. An e-learning training package and toolkit providing a comprehensive description of delivery of the STAR care pathway is available from the NHS Learning Hub [14, 15].

In the current study, we aimed to follow up STAR trial participants at a median of four years from randomisation to examine the longer-term clinical- and cost-effectiveness of the STAR care pathway for people with early chronic pain after TKR.

## Methods

### Patient and public involvement

Patient and Public Involvement (PPI) work enabled lived experience to inform follow-up of participants. The study benefitted from the continued input of the Patient Experience Partnership in Research (PEP-R) group, an experienced group of people with musculoskeletal conditions and/or experience of joint replacement surgery, who had been involved with the STAR trial from its conceptualisation. To ensure the inclusion of the perspectives of people with experience of chronic pain after TKR, participants in the STAR trial who were randomised to the intervention arm were invited to join a dedicated project forum following completion of the four-year follow-up questionnaire. The groups convened at key timepoints over the duration of the study to review and advise on the study approach processes, refine patient-facing documentation, and discuss appropriate channels for dissemination. When necessary, the PPI lead also met with patient members individually. A patient-partner was a member of the Programme Steering Committee, providing helpful oversight from the patient perspective.

### Study design

This is a longer-term follow-up of the STAR trial, which recruited participants from eight NHS hospitals in England and Wales. Trial participants were adults who underwent primary total knee replacement for osteoarthritis and reported pain in their replaced knee at three months after surgery, measured by the Oxford Knee Score (OKS) pain component [6]. The trial was registered (ISRCTN92545361) and results from the one-year follow-up are published [13]. The final follow-up in the original trial protocol was at 12 months after randomisation and was completed in June 2020 [10]. All participants consented to be contacted about participation in future research. Participants were sent a plain English summary of the results in December 2020, which advised that they may be contacted again about completing an additional questionnaire at approximately four years after they first enrolled in the trial. Separate protocol and ethics applications were prepared for the follow-up study. A favourable ethical opinion was issued by South Central – Oxford C Research Ethics Committee and study approval was obtained from the Health Research Authority (21/SC/0098, IRAS 296,241).

### Participants

Local NHS staff checked the hospital records of the 337 participants who were active in the trial at 12 months after randomisation (15 months after surgery) to confirm current address and vital status before sending an introduction letter. Two weeks later, a study pack was sent containing an approach letter, patient information booklet for the follow-up study, questionnaire, consent form (which included consent to obtain data from the patient’s medical records), and a postage-paid return envelope. An individually wrapped teabag was provided in each pack as a non-monetary incentive for participation; this was also provided in the main trial [13, 16].

### Outcomes

We collected patient-reported outcomes from all participants at a single timepoint, at a median of four years after randomisation, starting from April 2021 to January 2023 (mean 3.91 years, range 3.42 to 4.44 years). A 22-month window for data collection was available for a range of 32 months of timepoints. The four-year timepoint (follow-up due after randomisation) was 25 October 2020 for the first participant to 31 May 2023 for the final participant.

Outcomes collected in the trial were repeated for this four-year follow-up. The co-primary outcomes were self-reported pain severity and pain interference with daily living, measured by the Brief Pain Inventory (BPI) [17]. There is good evidence of the validity, reliability and sensitivity to change for the pain intensity and pain interference items of the BPI [18]. This self-report pain measure is recommended for use in clinical trials by the Initiative on Methods, Measurement and Pain Assessment in Clinical Trials (IMMPACT) [19]. It assesses pain severity through four items: worst, least, average and current pain; and pain interference through seven items about daily activities: general activity, mood, walking, normal work, relations with others, sleep, and enjoyment of life. Participants were asked to complete the BPI in relation to their operated knee.

Secondary outcomes were the: Oxford Knee Score (OKS) [20]; PainDETECT [21]; Douleur Neuropathique 4 (DN-4) [22]; Hospital Anxiety and Depression Scale (HADS) [23]; Pain Catastrophising Scale (PSC) [24]; Possible Solutions to Pain Questionnaire (PaSol) [25]; Self-Administered Patient Satisfaction Scale (PSS) [26]; single-item questions on pain frequency during past 24 h and 4 weeks; ICEpop CAPability measure for Adults (ICECAP-A) [27]; EQ-5D-5 L [28]; Short Form-12 (SF-12) [29]; and body diagram to assess widespread chronic pain.

Informatics Departments of the eight trial sites provided anonymised electronic hospital informatics coding data for each participant covering the period 12 to 48 months after randomisation. For inpatient stays and day cases, these included ICD10 (International Statistical Classification of Diseases), OPCS4 (International Classification of Interventions and Procedures), HRG (Health Resource Group) codes, admission and discharge dates. For outpatient visits, including procedures, radiology and Accident and Emergency attendances, data were obtained in the form of service codes, HRG/Currency codes and attendance dates.

The outcome for the economic evaluation was the Quality-adjusted life year (QALY). The EQ-5D-5 L was used to calculate QALYs as it had been shown in the original study to be sensitive in relation to improvement overtime in both arms, confirming previous research [13, 30]. To maintain consistency with the original trial, the van Hout mapping algorithm was used throughout to derive utility values from the EQ-5D-5 L questionnaire completed in the original trial and at the longer-term follow-up [31]. QALYs were then calculated using the area under the curve approach, taking into account deaths that had occurred from the end of the initial study and confirmation of vital status.

### Sample size and statistical analyses

Assuming attrition of 25% from those (337) participants still enrolled in the trial at 12 months after randomisation, a sample size of 253 for the longer-term follow-up would have 80–90% power to detect differences in the range 0.94–1.09 points on the BPI severity scale, using the observed standard deviation (SD) of 2.5 and a two-sided 5% significance level. With this SD, these detectable differences are equivalent to 0.38–0.43 SDs. The 2:1 intervention:control randomisation ratio means the projected sample sizes were 169:84.

All analyses were conducted in Stata version 17.0. Following an update to the STAR CONSORT participant flow diagram and descriptive statistics exploring any additional attrition, the primary analysis for this study was a comparison of the two treatment groups (as randomised) according to the co-primary outcomes, pain severity and pain interference as measured by the BPI. As well as descriptive statistics and plots, this involved linear regression models adjusting for baseline values of the outcomes and minimisation/stratification variables as fixed effects, presenting estimates, confidence intervals and p-values.

Sensitivity analyses considered a multiple imputation chained estimation (Stata command mi impute chained) based on missing at random assumption, and further analyses making the most extreme assumptions about missing data across the two arms (that is, in the first case, assuming the worst possible outcome for the intervention arm, and the best for the control arm, and in the second case, assuming the best possible outcome for the control arm, and the worst possible outcome for the intervention arm.

Similarities and differences at trial baseline between participants who provided four year follow-up data and those who did not were examined.

### Cost-effectiveness analysis

The cost-effectiveness analysis was from an NHS secondary care perspective with a four-year time horizon. The groups were compared as randomised. Inpatient stays, outpatient visits (including physiotherapy and OT appointments), procedures and Emergency Department attendances clinically judged (by NH, blinded to treatment arm) to be related to the patient’s TKR or ongoing pain in their knee were included in the analysis. NHS reference costs for 2020–2021 were used to value the resource use data obtained 12 to 48 months after randomisation [32]. Costs based on the hospital informatics data obtained 0–12 months from randomisation were inflated using the NHS cost inflation index (NHSCII) [33]. The cost of the original intervention was included in the calculation of the overall cost. Costs and QALYs accruing after one year from randomisation in the economic evaluation were discounted at 3.5%, as in the National Institute for Health and Care Excellence (NICE) reference case [34]. As in the original trial a multiple imputed dataset was used for the main analysis. Multiple imputation using chained equations and predictive mean matching was used. The imputation model, which was run by treatment group, included age, sex, study site, baseline parameters and cost data from the first 12 months of the trial. Forty-eight imputations were used and were combined using Rubin’s rules. Incremental costs and QALYs were estimated using a seemingly unrelated regression model. The outputs from the regression were used to estimate adjusted mean costs and QALYs, their between-group differences, the incremental net monetary benefit statistic (calculated at the standard NICE willingness-to-pay threshold of £20,000 per QALY) and the cost-effectiveness acceptability curve (CEAC). CEACs are used to explore uncertainty, they depict the probability that the intervention is cost-effective over a range of willingness-to-pay values for a QALY. Additionally, to explore the admission costs over time, generalised linear model (GLM) regression was used to estimate the mean adjusted costs and the difference in costs by arm.

Sensitivity analyses were used to address uncertainty. Different discount rates were used for costs and outcomes (1.5% and 5%). The base case analysis had used short stay (less than two days) costs and long stay costs (two days or more) to value the inpatient stays. This was altered to a value of an elective inpatient stay in the 2020-21 NHS reference costs. Finally, to explore assumptions in relation to missing data the following approach was taken. For each arm separately, a weighting factor was calculated by dividing the 12-month costs and utilities of those who did not complete the longer-term follow-up questionnaire with those who did. The weighted mean cost/utility was then used to impute missing longer-term follow-up cost and utility data.

## Results

### Participant flow

**Fig. 1:**
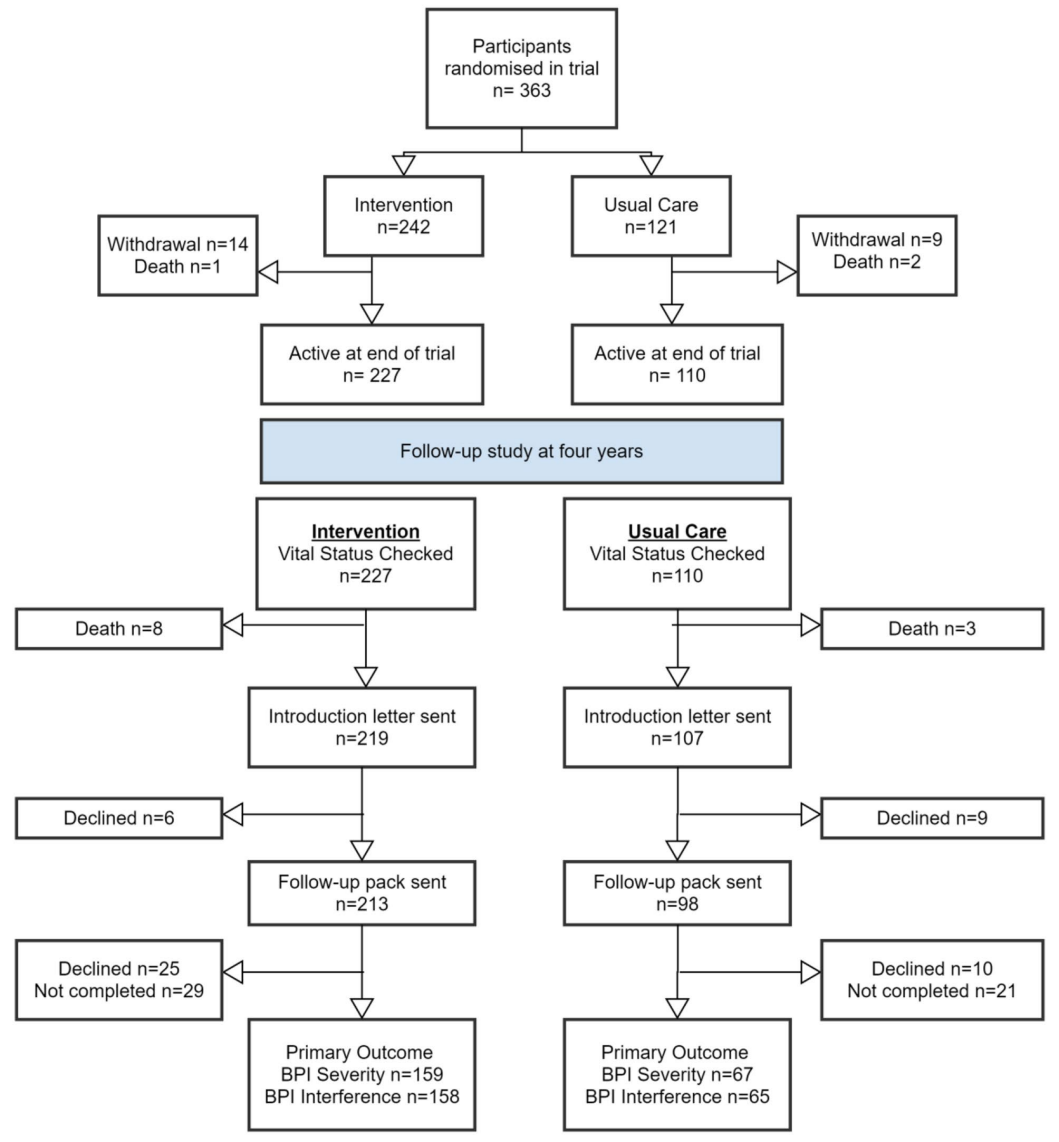
Extended CONSORT for participant flow from randomisation follow-up at four years

The STAR trial included 363 participants randomly allocated to intervention or usual care and 313 participants provided data at the 12-month follow-up timepoint [13]. However, at the 12-month follow-up, 337 participants were still enrolled on the trial. Even though 24/337 (7%) had not provided 12-month data, these participants had not withdrawn from the trial and therefore were included in the potential sample for this follow-up study (Fig. 1). Of these 337, vital status was confirmed at four years for 326 participants (97%), who were sent invitation letters. Of these, 100/326 (37%) were ‘non-responders’: 50/326 (15%) declined to take part and a further 50/326 (15%) did not reply to the invitation letter or were not contactable. Of the 50 who declined to take part, 32 provided reasons for declining participation. Reasons were reviewed and coded independently by two team members (WB and VW). These included: other commitments (n = 13, 41%), other health conditions (n = 11, 34%), did not want to complete more questionnaires (n = 3, 9%), study not perceived as relevant (n = 2, 6%), not interested (n = 2, 6%) and did not want more treatment (n = 1, 3%). The follow-up study comprised a single participant completed questionnaire and did not involve any more treatment.

Overall, 226/337 (67%) of those still enrolled in the trial at 12 months provided follow-up data at four years after randomisation and gave consent for extraction of information from their medical records; of these, 159 (70%) had received the intervention and 67 (30%) had usual care. This distribution was broadly reflective of the 2:1 randomisation ratio, albeit with a higher attrition rate (41%) in the usual care arm than in the intervention arm (30%) over time.

Median participant age at recruitment was 67 years (inter-quartile range 61–73), 217/363 (60%) were female and 335/363 (92%) were White. There were no substantial imbalances in participant characteristics between treatment arms at baseline; these data have been published [12, 13].

### Characteristics of responders and non-responders

Similarities and differences at trial baseline between characteristics, outcome scores and utility values of participants who provided four year follow-up data and those who did not are provided in additional file 1. Values were similar in patients who received usual care. However, in the intervention group, patients who completed the primary outcomes at a median of four years after randomisation had better values than those who did not complete this measure, suggesting non-random missing, which may (though may not) show results more favourable to the intervention.

**Fig. 2:**
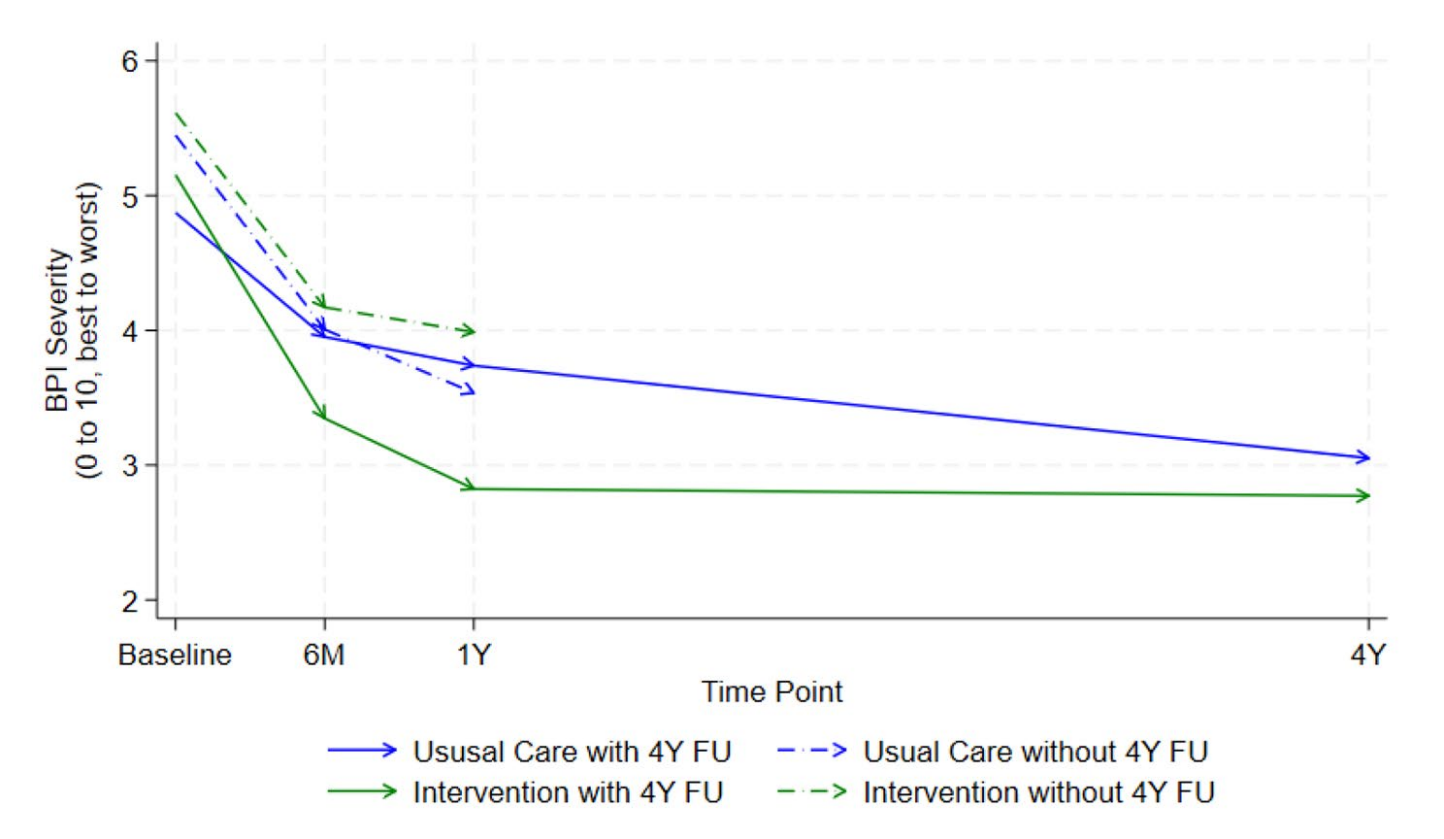
Mean BPI severity over time by trial arm and response to the four-year follow-up

**Fig. 3:**
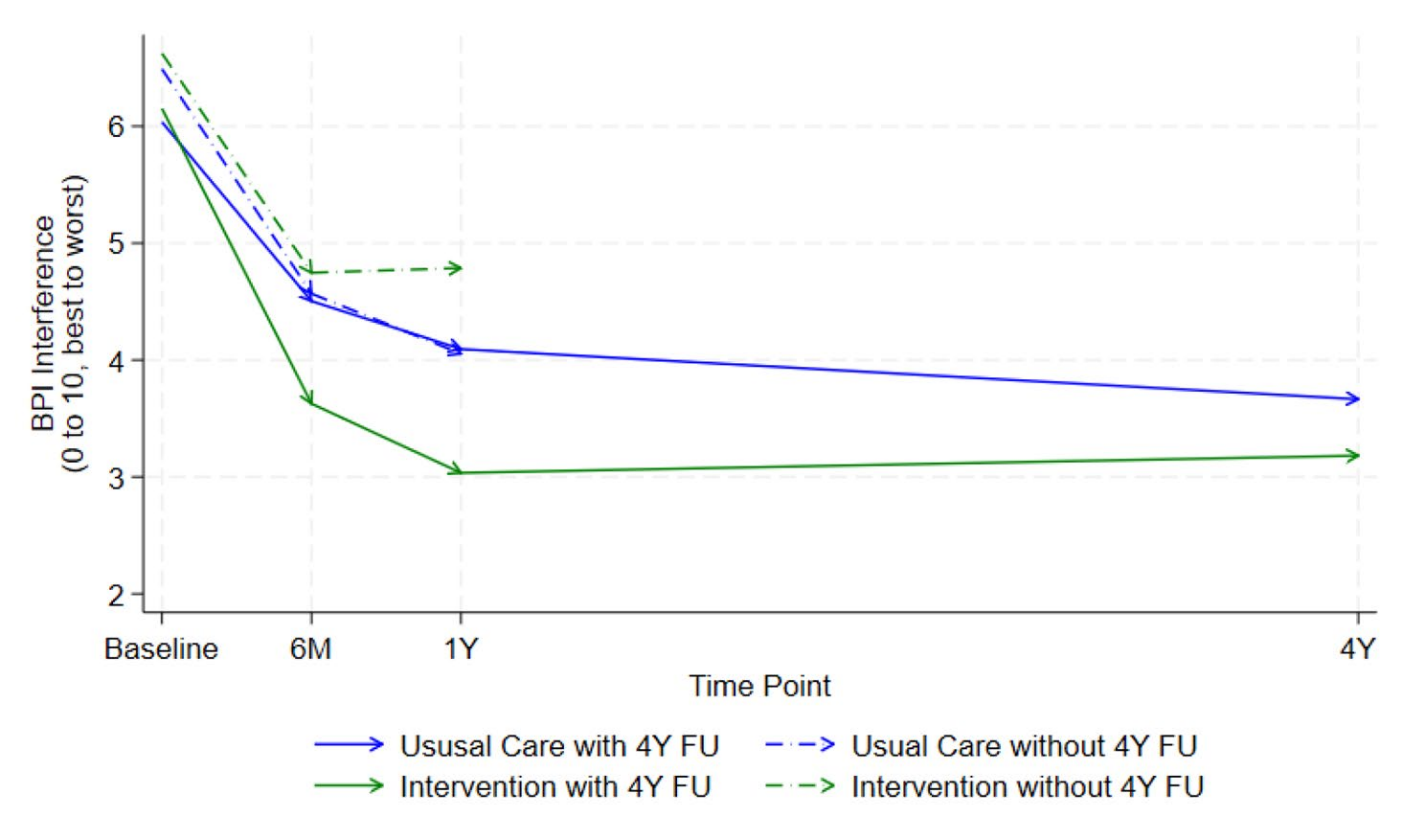
Mean BPI interference over time by trial arm and response to the four-year follow-up

Figures depicting the difference in means over time for the responders vs. non-responders for the two primary outcomes by arm suggest that the data is not missing at random (Figs. 2 and 3). Individual participant changes from baseline to four years in primary outcomes are provided in additional file 1.

**Fig. 4:**
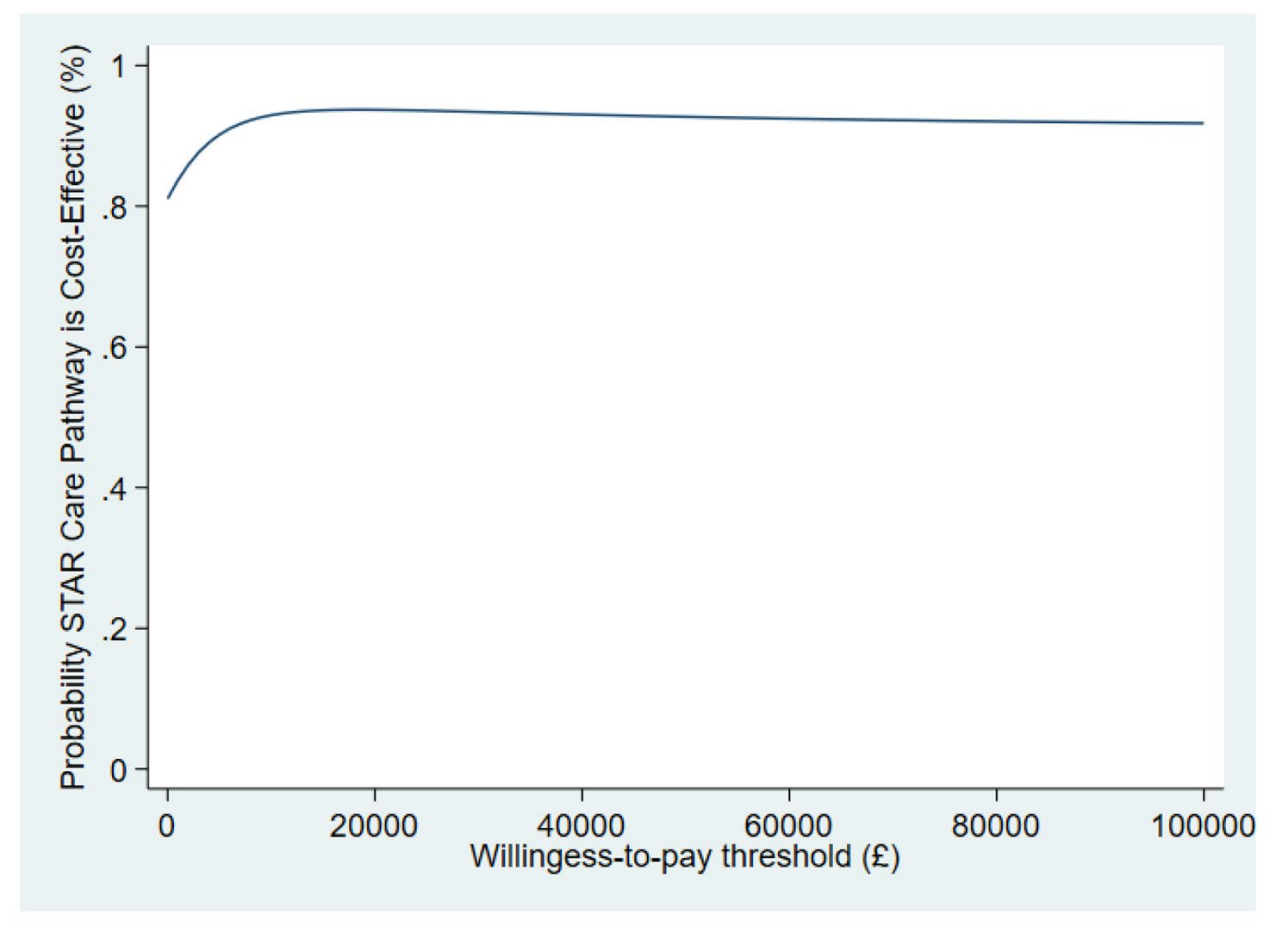
Cost effectiveness acceptability curve showing the probability that the STAR care pathway is cost-effective at different willingness-to-pay thresholds

Differences between the responders vs. non-responders to the four year follow-up were also seen in the EQ-5D-5 L. Baseline utility values were similar in patients who received usual care at 12 months. However, in the intervention group, patients who completed the EQ-5D-5 L questionnaire at a median of four years after randomisation had higher (baseline) utility values than those who did not complete this measure.

### Pain outcomes at four years

Pain severity data as measured by BPI were available for all participants who responded to the longer-term follow-up. The response rate was 62% (226/363). Pain interference data were available for all but two usual care participants and one intervention participant, with a response rate of 61% (223/363).

At four years, the adjusted between-group difference in means for the BPI pain severity score was − 0.42 (95% CI (-1.07, 0.23); *p* = 0.20) and − 0.64 (95% CI -1.41, 0.12); *p* = 0.10) for the pain interference score. Figures depicting the difference in means over time for responders versus non-responders, and by treatment arm are provided in Table 1.

**Table 1:** Primary and selected secondary outcomes at baseline, one- and four-year follow-up

		Usual Care		Intervention				
	(Range, direction)	N (110)	Mean (SD)	N (227)	Mean (SD)	Intervention effect	95% CI	***p*** value
**BPI Severity - BL**	(0 to 10, best to worst)	110	5.1 (1.6)	227	5.3 (1.7)			
**BPI Severity − 1Y**	(0 to 10, best to worst)	100	3.7 (2.5)	212	3.1 (2.4)	-0.65	(-1.17, -0.13)	0.014
**BPI Severity − 4Y**	(0 to 10, best to worst)	67	3.1 (2.4)	159	2.8 (2.6)	-0.42	(-1.07, 0.23)	0.202
**BPI Interference - BL**	(0 to 10, best to worst)	110	6.2 (2.0)	227	6.3 (1.9)			
**BPI Interference − 1Y**	(0 to 10, best to worst)	100	4.1 (2.9)	213	3.5 (2.8)	-0.68	(-1.28, -0.08)	0.026
**BPI Interference − 4Y**	(0 to 10, best to worst)	65	3.7 (3.0)	158	3.2 (3.0)	-0.64	(-1.41, 0.12)	0.099
**Oxford Knee Score - BL**	(0 to 48, worst to best)	110	18.9 (5.3)	227	18.0 (6.0)			
**Oxford Knee Score − 1Y**	(0 to 48, worst to best)	93	27.2 (9.7)	201	28.4 (10.2)	2.10	(-0.13, 4.33)	0.064
**Oxford Knee Score − 4Y**	(0 to 48, worst to best)	64	27.7 (11.8)	158	29.9 (11.3)	2.93	(-0.02, 5.89)	0.052
**PainDETECT - BL**	(–1 to 38, best to worst)	110	17.2 (6.9)	227	18.6 (6.7)			
**PainDETECT − 1Y**	(–1 to 38, best to worst)	94	12.7 (7.5)	198	12.9 (7.7)	-0.31	(-2.07, 1.44)	0.725
**PainDETECT − 4Y**	(–1 to 38, best to worst)	66	11.1 (7.7)	159	11.5 (8.5)	-0.43	(-2.51, 1.65)	0.686
**HADS** **Anxiety - BL**	(0 to 21, best to worst)	110	7.1 (4.6)	227	7.8 (4.5)			
**HADS Anxiety − 1Y**	(0 to 21, best to worst)	92	6.0 (4.7)	198	6.0 (4.4)	-0.04	(-1.10, 1.02)	0.941
**HADS Anxiety − 4Y**	(0 to 21, best to worst)	66	6.6 (4.6)	156	6.6 (5.1)	-0.66	(-1.76, 0.44)	0.238
**HADS Depression - BL**	(0 to 21, best to worst)	110	7.3 (3.9)	227	7.9 (4.1)			
**HADS Depression − 1Y**	(0 to 21, best to worst)	90	6.1 (4.3)	197	6.0 (3.8)	-0.31	(-1.23, 0.61)	0.511
**HADS Depression − 4Y**	(0 to 21, best to worst)	66	6.5 (4.6)	156	6.3 (4.2)	-0.45	(-1.44, 0.54)	0.373

Note: The “intervention effect” is intervention minus usual care, adjusted for baseline BPI Severity and Interference, and site. Secondary outcomes are adjusted for their corresponding baseline values.

Analysis making the most extreme assumptions about missing data across the two arms gave different results in direction as well as magnitude, though multiple imputation analyses (assuming the data is missing at random) led to attenuations of about 0.2 points from the above estimates of effect on the two co-primary outcomes. The reduced mice model included BPI severity and interference scores at all time points in chain. Site and baseline BPI severity scale, BPI interference scale, OKS, PainDETECT and HADS scores were included in the prediction model. The full mice model includes all primary and secondary outcomes (with the exception of chronic widespread pain) at all follow up points, computed in chain, and site, gender and baseline BPI severity, and interference, OKS, PainDETECT and HADS, baseline chronic widespread pain, and SF12.

OKS scores were higher in the intervention group than with usual care at four years but were not significant (mean between-group difference 2.93 [95% CI -0.02 to 5.89]; *p* = 0.052). The remaining secondary outcome measure did not reveal any addition effects. Secondary outcome results are reported in additional file 2.

### Cost-effectiveness analyses

Secondary care resource use for the STAR follow-up period was only available for the 226 participants who had consented to have information extracted from their medical records. Throughout the four-year time horizon (Table 2) the mean number of admissions was higher in the usual care arm compared to the intervention arm (0.76 versus 0.4). The mean number of admissions in the usual care arm were almost double those of the intervention arm up to one year after randomisation (0.25 versus 0.13) and also over longer follow-up, to four years after randomisation (0.49 versus 0.26). The number of outpatient visits were similar, except for outpatient speciality appointments, the usual care arm had slightly more events in the other outpatient categories.

**Table 2:** Resource use during the STAR trial and follow-up at four years

	Usual Care			Intervention		
Variable	N (110)	Mean	[95% CI]	N (227)	Mean	[95% CI]
**Number of Day case admissions**						
**STAR (0–1 year)**	110	0.05	(0.00, 0.10)	227	0.06	(0.03,0.09)
**STAR FU (1–4 Years)**	67	0.30	(0.16,0.43)	159	0.12	(0.06,0.18)
**4 Year Total (0–4 Years)**	67	0.37	(0.23,0.52)	159	0.17	(0.10,0.24)
**Number of Short stay admissions (< 2 days)**						
**STAR (0–1 year)**	110	0.07	(0.02,0.13)	227	0.03	(0.01,0.05)
**STAR FU (1–4 Years)**	67	0.01	(-0.01,0.04)	159	0.03	(0.00,0.05)
**4 Year Total (0–4 Years)**	67	0.10	(0.01,0.20)	159	0.06	(0.02,0.09)
**Number of Long stay admissions (≥ 2 days)**						
**STAR (0–1 year)**	110	0.13	(0.05,0.20)	227	0.05	(0.02,0.08)
**STAR FU (1–4 Years)**	67	0.18	(0.06,0.30)	159	0.12	(0.06,0.18)
**4 Year Total (0–4 Years)**	67	0.28	(0.14,0.42)	159	0.17	(0.09,0.25)
**Number of Total admissions**						
**STAR (0–1 year)**	110	0.25	(0.13,0.38)	227	0.13	(0.09,0.18)
**STAR FU (1–4 Years)**	67	0.49	(0.30,0.69)	159	0.26	(0.17,0.36)
**4 Year Total (0–4 Years)**	67	0.76	(0.50,1.03)	159	0.40	(0.28,0.52)
**Number of Outpatient speciality appointments**						
**STAR (0–1 year)**	110	7.43	(5.95,8.90)	227	7.25	(6.25,8.24)
**STAR FU (1–4 Years)**	67	6.93	(4.99,8.86)	159	7.58	(6.21,8.96)
**4 Year Total (0–4 Years)**	67	15.37	(12.06,18.69)	159	14.84	(12.54,17.13)
**Number of Outpatient procedure appointments**						
**STAR (0–1 year)**	110	0.04	(0.00,0.07)	227	0.13	(0.02,0.23)
**STAR FU (1–4 Years)**	67	0.16	(0.03,0.30)	159	0.21	(0.12,0.30)
**4 Year Total (0–4 Years)**	67	0.21	(0.07,0.35)	159	0.31	(0.17,0.44)
**Number of Outpatient radiology appointments**						
**STAR (0–1 year)**	110	0.81	(0.55,1.07)	227	0.65	(0.48,0.82)
**STAR FU (1–4 Years)**	67	0.81	(0.47,1.14)	159	0.84	(0.62,1.06)
**4 Year Total (0–4 Years)**	67	1.42	(0.92,1.92)	159	1.44	(1.10,1.78)
Number of Accident and Emergency attendances*						
**STAR (0–1 year)**	109	0.15	(0.05,0.25)	225	0.14	(0.09,0.20)
**STAR FU (1–4 Years)**	61	0.38	(0.19,0.57)	149	0.40	(0.22,0.57)
**4 Year Total (0–4 Years)**	61	0.46	(0.24,0.68)	149	0.55	(0.35,0.75)

*Two sites do not have an A&E department so the total numbers of patients for each group are different

Using a multiple imputed dataset for the 337 participants showed that the adjusted long stay admission costs were still the main cost driver for differences in NHS costs, in favour of the intervention arm (Table 3).

**Table 3:** Inpatient costs

	Usual Care			Intervention			Intervention-Usual Care	
Variable	N	Mean	[95% CI]	N	Mean	[95% CI]	Mean diff.	[95% CI]
**Costs of Day case admissions**								
**STAR (0–1 year)**	110	32	(-23, 87)	227	72	(33,110)	40	(-28, 107)
**STAR FU (1–4 Years)**	110	287	(160, 414)	227	153	(74, 231)	-135	(-287, 17)
**4 Year Total (0–4 Years)**	110	319	(180, 458)	227	224	(137, 312)	-95	(-262, 72)
**Costs of Short stay admissions (< 2 days)**								
**STAR (0–1 year)**	110	119	(49, 189)	227	41	(-8, 90)	-78	(-163, 8)
**STAR FU (1–4 Years)**	110	34	(-42, 110)	227	58	(0, 115)	24	(-72, 120)
**4 Year Total (0–4 Years)**	110	152	(46, 259)	227	99	(22, 176)	-54	(-186, 78)
**Costs of Long stay admissions (≥ 2 days)**								
**STAR (0–1 year)**	110	1133	(648, 1618)	227	396	(59,733)	-737	(-1328, -145)
**STAR FU (1–4 Years)**	110	2180	(629, 3730)	227	1841	(779, 2902)	-339	(-2235, 1557)
**4 Year Total (0–4 Years)**	110	3313	(1670, 4955)	227	2237	(1111, 3364)	-1075	(-3084, 933)
**Total admission costs**								
**STAR (0–1 year)**	110	1283	(786, 1781)	227	509	(164,855)	-744	(-1381, -167)
**STAR FU (1–4 Years)**	110	2501	(921, 4081)	227	2051	(980, 3122)	-450	(-2379, 1479)
**4 Year Total (0–4 Years)**	110	3784	(2106, 5462)	227	2560	(1419, 3701)	-1224	(-3272, 825)

The difference had attenuated over time from -£737 in the first year after randomisation to -£339 in years one-four, culminating in an overall cost difference of -£1075 (-£3084, £933) (Table 4).

**Table 4:** Cost-effectiveness results at 4 years

		Adjusted Costs	Adjusted QALYs	Incremental costs	Incremental QALYs	Incremental NMB at £20,000/QALY	Probability cost-effective at £20,000
	N	Mean £ (95% CI)	Mean (95% CI)	Mean £ (95% CI)	Mean (95% CI)	Mean £ (95% CI)	
**Intervention**	227	4982 (3727,6237)	2.60 (2.49,2.70)				
**Usual Care**	110	5983 (4164,7801)	2.47 (2.31,2.63)				
**Intervention vs. usual care**				-1001 (-3238,1236)	0.13 (-0.06,0.32)	3525 (-990, 8039)	0.94

All variables are adjusted for hospital site and baseline BPI subscores. Additionally, QALYs are adjusted for baseline utility

**Table 5:** Sensitivity analyses (cost effectiveness)

	N	Adjusted Costs Mean £ (95% CI)	Adjusted QALYs Mean (95% CI)	Incremental costs Mean £ (95% CI)	Incremental QALYs (95% CI)	Incremental NMB at £20,000/QALY Mean (95% CI)	Probability cost-effective at £20,000
**Discounted at 1.5%**							
**Intervention (1.5%)**	227	5073 (3807,6340)	2.65 (2.54,2.75)				
**Usual Care (1.5%)**	110	6120 (4472,7768)	2.53 (2.36,2.69)				
**Intervention vs. usual care (1.5%)**				-1046 (-3099, 1006)	0.12 (-0.08,0.32)	3458 (-1025, 7940)	0.93
**Discounted at 5%**							
**Intervention (5%)**	227	4869 (3707,6031)	2.55 (2.44,2.65)				
**Usual Care (5%)**	110	5982 (4184,7600)	2.42 (2.27,2.58)				
**Intervention vs. usual care (5%)**				-1023 (-3033, 987)	0.12 (-0.07,0.30)	3392 (-931, 7716)	0.94
**Elective inpatient stays**							
**Intervention**	227	4331 (3426,5236)	2.60 (2.48,2.70)				
**Usual Care**	110	5197 (3881,6513)	2.47 (2.32,2.63)				
**Intervention vs. usual care**				-866 (-2460, 728)	0.12 (-0.07,0.30)	3170 (-1012, 7351)	0.93
**Missing values**							
**Intervention**	227	4954 (4052, 5857)	2.54 (2.41,2.66)				
**Usual Care**	110	5876 (4639,7112)	2.45 (2.28,2.63)				
**Intervention vs. usual care**				-1007 (-2533, 519)	0.12 (-0.05,0.29)	3330 (-480, 7142)	0.96

This resulted in adjusted mean NHS costs (including the costs of the STAR care pathway) being -£1001 (95% CI -£3238, £1236) lower in the intervention arm. The adjusted difference in mean QALYs of 0.13 (95% CI -0.06, 0.31) also favoured the intervention arm. This led to an incremental net monetary benefit of £3,525 (95% CI -£990 to £8039) at a £20,000/QALY willingness-to-pay threshold, and the probability of being cost-effective at this threshold of 0.94 (Fig. 4). There is high probability of the STAR care pathway intervention being cost-effective at all willingness-to-pay thresholds.

The sensitivity analyses showed the initial analyses to be robust (Table 5) with the probability of the intervention being the cost-effective option being 0.93 or above for all the analyses as the willingness-to-pay threshold of £20,000 per QALY.

## Discussion

At four years after delivery, the STAR care pathway remained a cost-effective treatment to improve pain outcomes for people with chronic pain at three months after TKR. The point estimates of effect among those reporting pain outcomes at four years are consistent with findings at 12 months (BPI pain severity − 0.42 at four years versus − 0.65 at one year, BPI pain interference − 0.64 versus − 0.68 at one year), therefore any longer-term disbenefit from the care pathway can be ruled out.

Cost savings in terms of NHS long stay admissions were £737 in the first year. Further costs savings of £339 between one year to four years after delivery suggest there are no bounce back effects on costs over the longer term. There are currently no other evidence-based treatment options for patients with chronic post-surgical pain after TKR. Implementation of the STAR care pathway presents a clinically important and cost-effective treatment option for those patients who will present with chronic post-surgical pain three months after TKR. We have since developed an e-learning training package and delivery toolkit to enable and support national implementation of the STAR care pathway in the NHS [14, 15].

The strength of evidence from the longer-term follow-up is limited by the lower response rates, with 86% (n = 313/363) of the randomised sample contributing data at one year and 67% (n = 226/337) of those enrolled at 12 months after randomisation contributing at four years. Our findings are therefore, based on two-thirds of the trial population. However, there were no substantial differences in measured baseline characteristics or utility values between participants who completed four-year follow-up questionnaires and those who did not. Any small differences between the two groups work against the intervention and have been adjusted for in the analysis. By four-year follow-up, it is over three years since most participants have had any form of intervention, and it is difficult to quantify the effects of any treatments subsequent to the STAR care pathway.

Surgical trials often have routine follow-up that improves attrition rates. Loss to follow-up in orthopaedic trials has been estimated to be 15% at three years [35]. However, few studies of non-surgical, rehabilitation-type interventions with longer-term follow-up are available in the literature. A trial of patients with knee osteoarthritis randomised to non-surgical treatments reported five-year follow-up rates of 78% in intervention and 72% in control groups [36]. A three-year follow-up of participants from a back pain treatment trial reported follow-up rates of 70% in both treatment arms [37]. Participants in the STAR trial originally consented to participate for one year and therefore did not expect to be asked to complete longer term follow-up. We have some reasons for declining from those who chose not to take part, although most chose not to give a reason. Participants withdrawing for non-effect or adverse events is common in chronic pain trials [38–41]. Here, we can speculate that although consent processes for the longer-term follow-up were robust and received research ethics approval, the participants’ original expectations may mean that those with ongoing pain and complications were less likely to return the follow-up questionnaire than those who had recovered. Although the attrition rate leads to an equivocal effect at four years in the context of limited power, and selection bias cannot be ruled out, the clinical effects do not appear to be entirely attenuated.

Community based NHS care and personal expenses were not collected for this follow-up study. The recall bias resulting from the long recall period (median 3 years) needed to obtain this information would have called into question the validity of these data. Further, in the original trial we had shown that inpatient stays were the main NHS cost driver and hence this was our focus in the follow-up study. A limitation of the study is therefore that the long-term effect on community based NHS care and personal expenses is unknown. In the original study the main cost driver from the patient perspective was hours of unpaid leave which was in favour of the intervention arm, therefore the follow-up results are potentially conservative in nature.

The higher number of inpatient admissions and their duration in the usual care arm continued in the longer-term follow-up. This translated into higher costs for the usual care arm and was the main source of the differences in costs between the treatment groups; QALYs remained higher in the intervention group compared to the conventional treatment group. Again, the results may have been influenced by the differential attrition rates between the trial arms, which would imply that the data were not missing at random. However, a sensitivity analysis which considered the likely biases, did not alter the original findings. From a secondary care NHS perspective, the conclusion that the intervention is the cost-effective option still holds within longer-term follow-up.

## Conclusions

In conclusion, the STAR care pathway, a clinically important and cost-effective intervention over one year for people with ongoing pain at three months after TKR, remains cost-effective at the longer-term follow-up of around four years. The magnitude of the longer-term benefits of the care pathway are less certain due to attrition of trial participants; however, there is a suggestion of some degree of sustained effects at four years in terms of clinical effectiveness, and clear evidence of sustained cost-effectiveness.

The STAR care pathway is currently the only evidence-based intervention for people with chronic pain after TKR. Implementation in usual care is needed to improve care and optimise outcomes.

### Electronic supplementary material

Below is the link to the electronic supplementary material.

Supplementary Material 1

## Data Availability

The data sets generated during the current longer-term follow-up study will be available in the University of Bristol Research Data Repository (https://data.bris.ac.uk/data/) within six months following final publication of this article. Access to the data will be restricted to ensure that data are only made available to bona fide researchers for ethically approved research projects, on the understanding that confidentiality will be maintained and after a data access agreement has been signed by an institutional signatory. Data sets generated during the trial are available in the University of Bristol Research Data Repository. Rachael Gooberman-Hill, Vikki Wylde, Wendy Bertram (2022): STAR Trial. 10.5523/bris.2upet8ppspfz8271zvveapirqj.

## Declarations

### STAR trial sites

Southmead Hospital, North Bristol NHS Trust. Princess Elizabeth Orthopaedic Centre, Royal Devon and Exeter NHS Trust. Llandough Hospital, Cardiff and Vale University Health Board. The Robert Jones and Agnes Hunt Orthopaedic Hospital NHS Trust. King’s Mills Hospital, Sherwood Forest Hospitals NHS Trust. Wrightington Hospital, Wrightington, Wigan and Leigh NHS Trust. Leicester General Hospital, University Hospitals of Leicester NHS Trust. The Royal Orthopaedic Hospital NHS Foundation Trust.

## Abbreviations

BPI: Brief Pain Inventory
DN-4: Douleur Neuropathique
HADS: Hospital Anxiety and Depression Scale
ICECAP-A: ICEpop CAPability measure for Adults
NHS: National Health Service
NICE: National Institute for Health and Care Excellence
OKS: Oxford Knee Score
PaSol: Possible Solutions to Pain Questionnaire
PEP-R: Patient Experience Partnership in Research
PCS: Pain Catastrophising Scale
PPI: Patient and Public Involvement
PSS: Patient Satisfaction Scale
QALY: Quality-adjusted life year
SF-12: Short Form-12
STAR: Support and Treatment After Replacement
TKR: Total Knee Replacement
