# Supplementary material for: The STAR care pathway for patients with chronic pain after total knee replacement: four-year follow-up of a randomised controlled trial
Source: BMC Musculoskelet Disord. 2023 Dec 16;24:972. doi: 10.1186/s12891-023-07099-x (PMC10725008; doi:10.1186/s12891-023-07099-x)
Supplement: Supplementary file 1 — Supplementary Material 1 [file 12891_2023_7099_MOESM1_ESM.docx]

**Additional file 1****: Baseline characteristics of responders and non-responders to four-year follow-up**

|  | **Responders** | | **Non-Responders** | |
| --- | --- | --- | --- | --- |
|  | **Usual Care (n=67)** | **Intervention (n=159)** | **Usual Care (n=54)** | **Intervention (n=83)** |
| **Median Age**, year (IQR) | 67 (60-73) | 67 (61-73) | 70 (63-75) | 67 (61-73) |
| **Gender** |  |  |  |  |
| Female | 40 (60%) | 93 (58%) | 36 (67%) | 48 (58%) |
| Male | 27 (40%) | 66 (42%) | 18 (33%) | 35 (42%) |
| **Marital Status** |  |  |  |  |
| Single | 7 (10%) | 9 (6%) | 4 (7%) | 5 (6%) |
| Married/partner | 44 (66%) | 109 (69%) | 37 (69%) | 61 (73%) |
| Divorced/separated | 8 (12%) | 14 (9%) | 5 (9%) | 8 (10%) |
| Widowed | 3 (4%) | 25 (16%) | 8 (15%) | 9 (11%) |
| Missing | 5 (7%) | 2 (1%) | 0 (0%) | 0 (0%) |
| **Living arrangement** |  |  |  |  |
| Alone | 8 (12%) | 37 (23%) | 14 (26%) | 19 (23%) |
| With husband/wife/partner | 46 (69%) | 109 (69%) | 36 (67%) | 62 (75%) |
| With somebody else | 8 (12%) | 10 (6%) | 3 (6%) | 1 (1%) |
| Other | 0 (0%) | 1 (1%) | 1 (2%) | 1 (1%) |
| Missing | 5 (7%) | 2 (1%) | 0 (0%) | 0 (0%) |
| **Ethnic group** |  |  |  |  |
| White | 59 (88%) | 150 (94%) | 50 (93%) | 76 (92%) |
| Mixed | 0 (0%) | 1 (1%) | 0 (0%) | 0 (0%) |
| Asian | 1 (1%) | 3 (2%) | 4 (7%) | 3 (4%) |
| Black | 1 (1%) | 1 (1%) | 0 (0%) | 3 (4%) |
| Other | 1 (1%) | 2 (1%) | 0 (0%) | 1 (1%) |
| Missing | 5 (7%) | 2 (1%) | 0 (0%) | 0 (0%) |
| **Education level** |  |  |  |  |
| Before normal school leaving age | 4 (6%) | 9 (6%) | 4 (7%) | 5 (6%) |
| At normal school leaving age | 32 (48%) | 82 (52%) | 29 (54%) | 51 (61%) |
| After college diploma or equivalent | 9 (13%) | 26 (16%) | 13 (24%) | 13 (16%) |
| After university degree | 2 (3%) | 10 (6%) | 0 (0%) | 3 (4%) |
| Postgraduate education | 8 (12%) | 10 (6%) | 4 (7%) | 2 (2%) |
| Other | 0 (0%) | 2 (1%) | 0 (0%) | 1 (1%) |
| Missing | 12 (18%) | 20 (13%) | 4 (7%) | 8 (10%) |
| **Outcomes** | Mean (SD) N | Mean (SD) N | Mean (SD) N | Mean (SD) N |
| BPI Severity | 4.9 (1.5) 67 | 5.2 (1.7) 159 | 5.5 (1.7) 54 | 5.5 (1.7) 83 |
| BPI Interference | 6.0 (1.9) 67 | 6.2 (1.9) 159 | 6.6 (1.9) 54 | 6.5 (2.0) 83 |
| Oxford Knee Score | 19.7 (4.7) 67 | 18.6 (5.7) 159 | 17.3 (5.8) 54 | 16.9 (6.6) 83 |
| PainDETECT | 16.7 (6.0) 67 | 18.1 (6.4) 159 | 18.7 (7.7) 54 | 19.3 (7.2) 83 |
| HADS Anxiety | 6.6 (3.9) 67 | 7.4 (4.4) 159 | 8.1 (5.3) 54 | 8.6 (4.6) 83 |
| HADS Depression | 7.1 (3.8) 67 | 7.4 (4.0) 159 | 7.8 (4.3) 54 | 9.0 (4.1) 83 |
| Utility Values: Mean adjusted QALYs (95% CI) | 0.48 (0.43 to 0.53) | 0.47 (0.44 to 0.50) | 0.44 (0.38 to 0.50) | 0.40 (0.35 to 0.46) |

**Additional file 2: Individual participant changes from baseline to four years in primary outcomes**

Individual participant changes from baseline in BPI severity


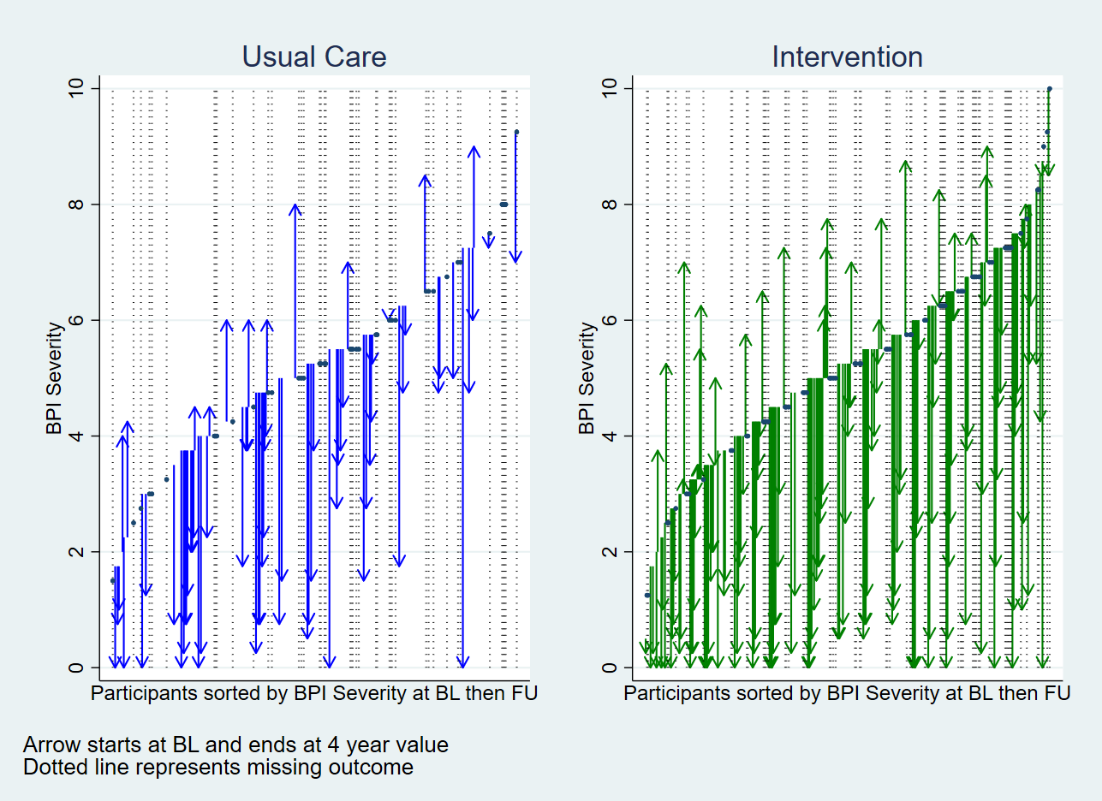


Individual participant changes from baseline in BPI Interference


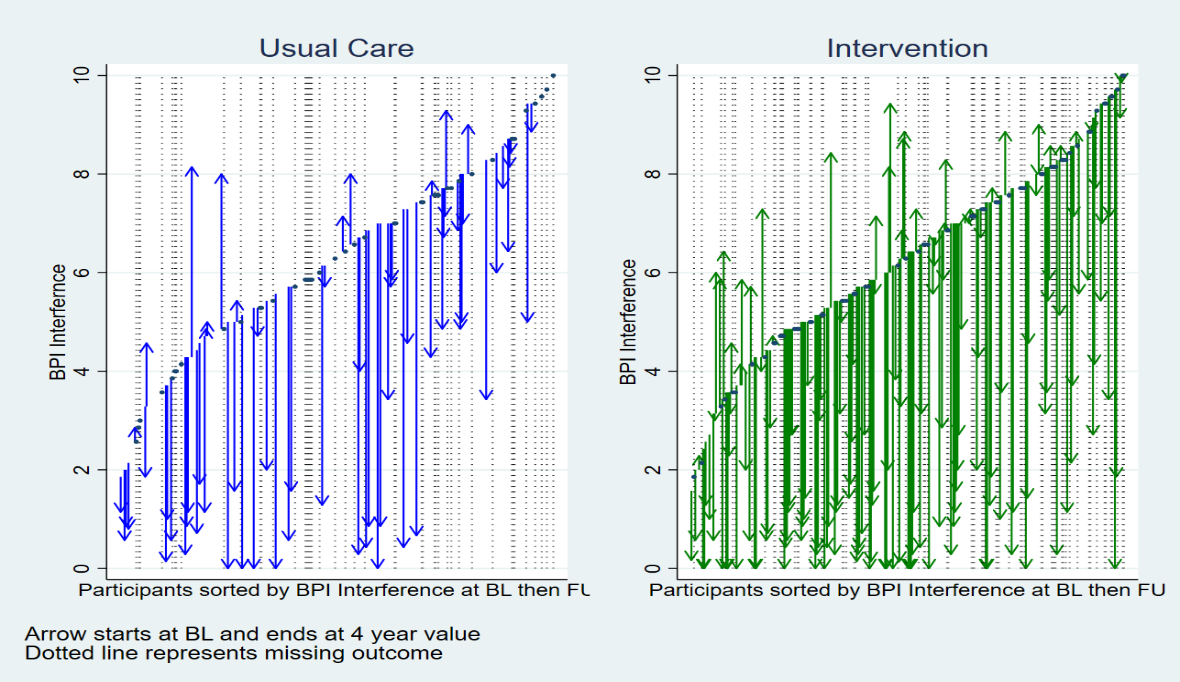


**Additional file 3: Secondary outcomes at baseline (BL) and four-year (4Y) follow-up for responders**

|  |  | **Usual Care** | | **Intervention** | |  |  |  |
| --- | --- | --- | --- | --- | --- | --- | --- | --- |
|  | **Range, Direction** | **N = 110** | **Mean, (SD)** | **N = 227** | **Mean, (SD)** | **Treatment effect** | **CI** | **p value** |
| Oxford Knee Score (BL) | (0 to 48, worst to best) | 64 | 19.6 (4.8) | 158 | 18.6 (5.7) |  |  |  |
| Oxford Knee Score (4Y) | (0 to 48, worst to best) | 64 | 27.7 (11.8) | 158 | 29.9 (11.3) | 2.93 | (-0.02, 5.89) | 0.052 |
| DN-4 (BL) | (0 to 7, best to worst) | 61 | 3.4 (1.6) | 155 | 3.7 (1.6) |  |  |  |
| DN-4 (4Y) | (0 to 7, best to worst) | 63 | 0.4 (0.3) | 156 | 0.4 (0.3) | -0.02 | (-0.10, 0.07) | 0.703 |
| PainDETECT (BL) | (–1 to 38, best to worst) | 66 | 16.6 (6.1) | 159 | 18.1 (6.4) |  |  |  |
| Pain Detect (4Y) | (–1 to 38, best to worst) | 66 | 11.1 (7.7) | 159 | 11.5 (8.5) | -0.43 | (-2.51, 1.65) | 0.686 |
| Pain Catastrophizing Scale (BL) | (0 to 52, best to worse) | 65 | 18.1 (12.4) | 146 | 18.9 (12.3) |  |  |  |
| Pain Catastrophizing Scale (4Y) | (0 to 52, best to worse) | 65 | 14.8 (15.2) | 147 | 13.4 (13.3) | -2.32 | (-5.88, 1.24) | 0.200 |
| PaSol: solving pain (BL) | (0 to 24, worst to best) | 66 | 17.1 (6.8) | 154 | 17.2 (5.7) |  |  |  |
| PaSol: solving pain (4Y) | (0 to 24, worst to best) | 66 | 12.8 (8.1) | 154 | 13.2 (8.2) | 0.23 | (-2.10, 2.57) | 0.845 |
| PaSol: meaningful life (BL) | (0 to 30, worst to best) | 66 | 22.3 (6.2) | 154 | 22.1 (5.5) |  |  |  |
| PaSol: meaningful life (4Y) | (0 to 30, worst to best) | 66 | 19.8 (8.9) | 154 | 19.2 (9.4) | -0.81 | (-3.55, 1.92) | 0.558 |
| PaSol: acceptance of pain (BL) | (0 to 18, worst to best) | 65 | 7.2 (5.0) | 152 | 7.7 (4.8) |  |  |  |
| PaSol: acceptance of pain (4Y) | (0 to 18, worst to best) | 66 | 10.0 (5.8) | 152 | 9.3 (6.0) | -0.73 | (-2.43, 0.97) | 0.399 |
| PaSol: belief in solution (BL) | (0 to 12, worst to best) | 65 | 8.7 (2.9) | 153 | 8.6 (3.1) |  |  |  |
| PaSol: belief in solution (4Y) | (0 to 12, worst to best) | 66 | 5.7 (4.3) | 154 | 5.5 (4.2) | -0.42 | (-1.67, 0.83) | 0.508 |
| Patient Satisfaction Scale (BL) | (25 to 100, worst to best) | 66 | 64.6 (18.1) | 156 | 64.9 (19.8) |  |  |  |
| Patient Satisfaction Scale (4Y) | (25 to 100, worst to best) | 66 | 4.8 (3.7) | 156 | 4.3 (3.8) | -0.58 | (-1.63, 0.48) | 0.284 |
| ICECAP-A (BL) | (–0·001 to 1, worst to best) | 65 | 0.8 (0.2) | 153 | 0.7 (0.2) |  |  |  |
| ICECAP-A (4Y) | (–0·001 to 1, worst to best) | 65 | 0.7 (0.2) | 154 | 0.8 (0.2) | 0.04 | (0.00, 0.09) | 0.063 |
| SF-12 Physical (BL) | (0 to 100, worst to best) | 66 | 34.7 (6.2) | 157 | 33.1 (6.6) |  |  |  |
| SF-12 Physical (4Y) | (0 to 100, worst to best) | 66 | 37.0 (10.7) | 157 | 36.9 (10.5) | 1.28 | (-1.48, 4.04) | 0.363 |
| SF-12 Mental (BL) | (0 to 100, worst to best) | 66 | 43.1 (10.6) | 157 | 43.2 (11.3) |  |  |  |
| SF-12 Mental (4Y) | (0 to 100, worst to best) | 66 | 47.9 (11.9) | 157 | 47.7 (12.4) | -0.19 | (-3.14, 2.75) | 0.896 |
| HADS Anxiety (BL) | (0 to 21, best to worst) | 66 | 6.6 (4.0) | 156 | 7.4 (4.4) |  |  |  |
| HADS Anxiety (4Y) | (0 to 21, best to worst) | 66 | 6.6 (4.6) | 156 | 6.6 (5.1) | -0.66 | (-1.76, 0.44) | 0.238 |
| HADS Depression (BL) | (0 to 21, best to worst) | 66 | 7.2 (3.8) | 156 | 7.5 (4.0) |  |  |  |
| HADS Depression (4Y) | (0 to 21, best to worst) | 66 | 6.5 (4.6) | 156 | 6.3 (4.2) | -0.45 | (-1.44, 0.54) | 0.373 |
